# New insights emerge as antibody repertoire diversification meets chromosome conformation

**DOI:** 10.12688/f1000research.17358.1

**Published:** 2019-03-28

**Authors:** Amy L. Kenter, Ann J. Feeney

**Affiliations:** 1 Department of Microbiology and Immunology, University of Illinois College of Medicine, Chicago, IL, 60612-7344, USA; 2 Department of Immunology and Microbiology, The Scripps Research Institute, La Jolla, CA, 92037, USA

**Keywords:** antibody genes, B cells, V(D)J recombination

## Abstract

Vast repertoires of unique antigen receptors are created in developing lymphocytes. The antigen receptor loci contain many variable (V), diversity (D), and joining (J) gene segments that are arrayed across very large genomic expanses and are joined to form variable-region exons. This process creates the potential for an organism to respond to large numbers of different pathogens. Here, we consider the underlying molecular mechanisms that favor some V genes for recombination prior to selection of the final antigen receptor repertoire. We discuss chromatin structures that form in antigen receptor loci to permit spatial proximity among the V, D, and J gene segments and how these relate to the generation of antigen receptor diversity.

## Introduction

**Figure 1.**
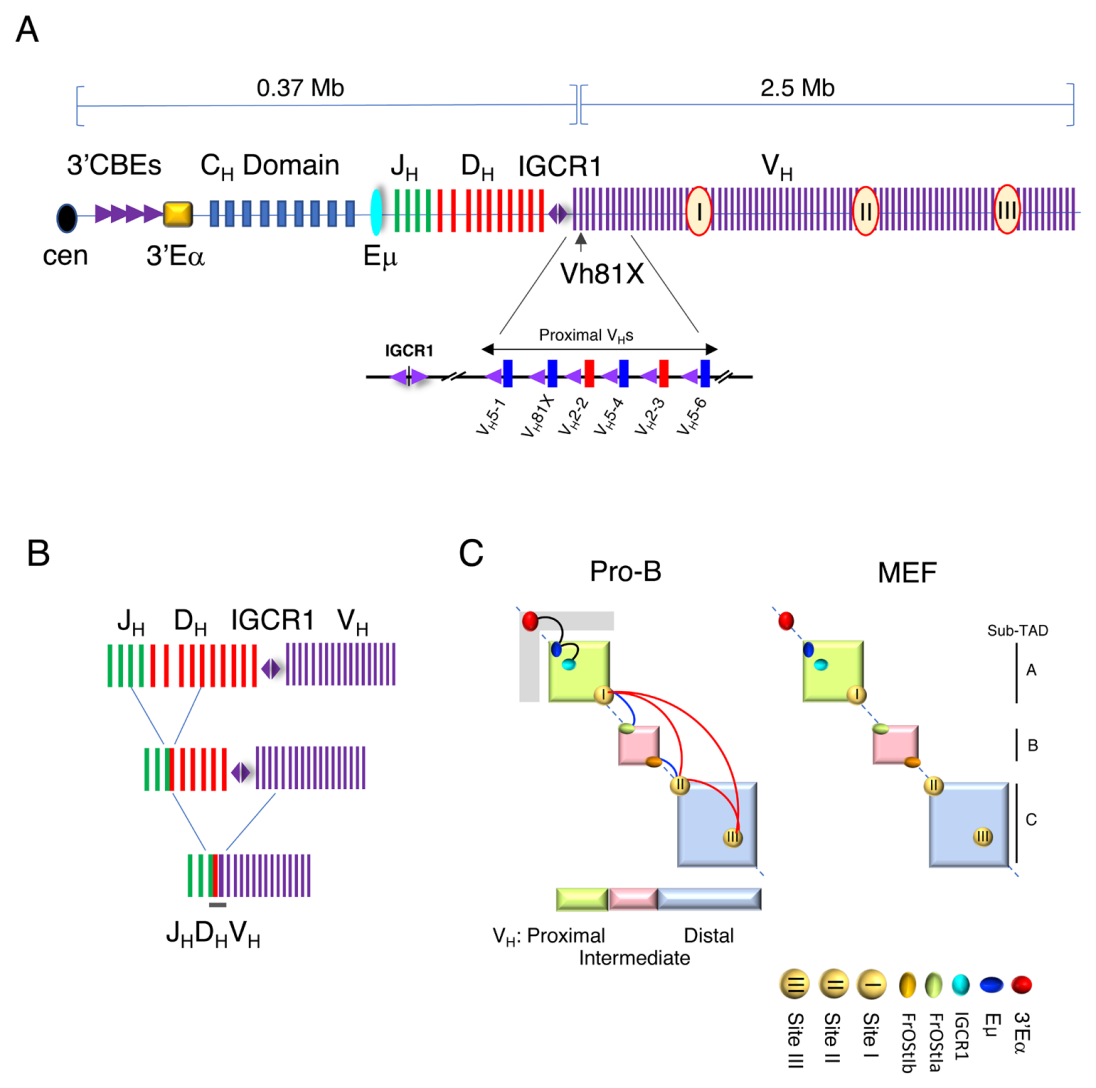
Overview of the Igh locus. The Igh locus spans 2.9 Mb and contains about 100 V _H_ gene segments. ( **A**) (Upper panel) Schematic diagram of the Igh locus showing the V _H_s, Ds, J _H_s, and C _H_ exons and regulatory elements (not to scale). The V _H_7183 and V _H_Q52 families—blue and red bars, respectively (lower panel)—are located at the D _H_J _H_-proximal end of the locus. Each D _H_J _H_-proximal V _H_ gene segment is paired with a recombination signal sequence (not shown) and a CTCF-binding element (CBE) (purple triangles). The CBE associated with the V _H_5-1 segment is non-functional (gray triangle). CBE orientation is indicated by the direction of the triangle. V _H_ gene segment names indicate their position along the locus. V _H_81X (V _H_5-2) is the original name of the second gene segment relative to intergenic control region 1 (IGCR1) and is used because it is well known by this nomenclature. The intermediate V _H_ segments include the V _H_S107 family along with nine smaller V _H_ families. At the 5′ end of the locus, the interspersed distal V _H_ segments are composed of the V _H_J558 and V _H_3609 families. Regulatory elements include intronic Eμ and 3′Eα super-enhancers and IGCR1, which is composed of two divergent CBEs. A cluster of at least nine CBEs is located at the 3′ boundary of the Igh locus and is adjacent to 3′Eα. The 3′CBEs and 3′Eα are referred to as the 3′ regulatory region (3′RR). Sites I, II, and III (red circles) engage in exceptionally long-range looping interactions and may mediate locus compaction. Sub-topologically associating domain (Sub-TADs) A, B, and C are indicated. ( **B**) Diagram of the stepwise process of V(D)J recombination. D-J rearrangement precedes V-DJ recombination. ( **C**) A schematic of the Igh TAD in pro-B cells that is subdivided into three sub-TADs A, B, and C. Looping interactions between Eμ:3′Eα and Eμ:IGCR1 (black arcs), Sites I and II, Sites II and III, Sites I to III (red arcs), Site I-FrOStIa, and Site II-FrOStIb (blue arcs) were detected and are not described here in detail ^1^.

**Figure 2.**
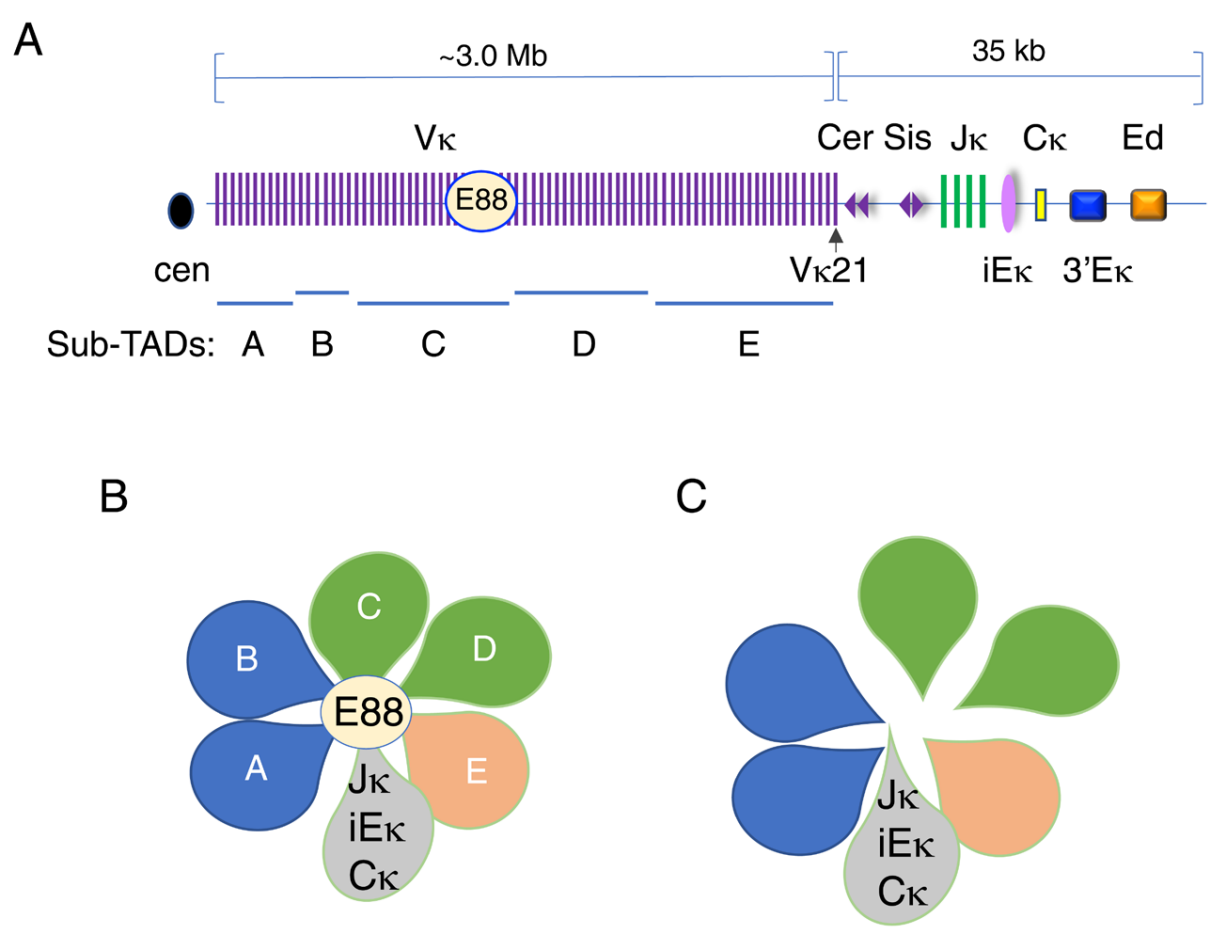
Three-dimensional conformation of the Igκ locus. The Igκ locus spans 3.2-Mb topologically associating domain (TAD) and contains about 120 functional Vκ gene segments. ( **A**) Schematic diagram of the Igκ locus showing the Vs, Js, and C exons and regulatory elements (not to scale). Regulatory elements include intronic Eκ (iEκ), 3′Eκ, Ed, and E88 elements. Contracting element for recombination (Cer) and silencer in the intervening sequence (Sis) are located between the V and J domains and are composed of CTCF-binding elements (CBEs) (purple triangles). The orientation of each CBE is indicated. The Igκ locus is subdivided into five sub-TADs (A–E) as indicated. ( **B**) Sub-TAD structure of the Igκ locus as determined by Hi-C ^2^. Each loop represents a sub-TAD that is labeled A–E. The regulatory region containing the Jκ genes, the three distal enhancers, and the constant region are in gray. ( **C**) Deletion of E88 results in untethering of sub-TADs C and D from the regulatory region.

In vertebrates, the adaptive immune response is capable of recognizing pathogens using antigen-specific receptors expressed on B and T lymphocytes. The B-cell receptor (BCR) is composed of two identical immunoglobulin (Ig) heavy chains (IgH) and two identical light chains (Igκ or Igλ). There are two lineages of T cells that are distinguished by the type of T-cell receptor (TCR) expressed. TCRαβ is encoded by the Tcra and Tcrb loci, whereas TCRγδ is encoded by the Tcrg and Tcrd loci. Antigen receptors are composed of variable (V) and constant (C) regions. The organization of the Igh and Igκ loci are schematically depicted ( Figure 1A and Figure 2A). Igh variable-region exons are produced by the joining of one each of the many variable (V), diversity (D), and joining (J) gene segments, whereas Igκ and Igλ are created by joining one each of the V and J gene segments, all by V(D)J recombination during lymphocyte development ( Figure 1B). V(D)J recombination is a stepwise process during which D _H_-to-J _H_ recombination occurs first followed by V _H_-to-D _H_J _H_ rearrangement. This process depends on the lymphocyte-specific V(D)J recombinase, RAG1/2, which recognizes recombination signal sequences (RSSs) that flank all V, D, and J gene segments ^3^. During V(D)J recombination, two RSSs adjacent to V, D, or J gene segments partner such that cleavage and rejoining occur. RAG1 contains endonuclease activity and targets the RSS, and RAG2 is recruited to the epigenetically modified histone 3 when it is trimethylated on lysine 4 ^3^. Within each antigen receptor locus, the RAG recombinase concentrates in the recombination center (RC) that is focused to the J segment–containing domain. Double-strand DNA breaks are generated at RSSs by RAG1/2, and the V, D, and J exons are joined together through non-homologous end joining ^4^.

Antigen receptor gene rearrangement is tightly regulated during lymphocyte development; in turn, lymphocyte development is strictly dependent on V(D)J recombination ^5, 6^. The composition and complexity of antigen receptor repertoires depend on the number of V, D, and J gene segments and the degree to which those segments are available for rearrangement. However, V gene usage in the pre-selected Igh repertoire is only quasi-random since it has been shown that V genes rearrange at very different intrinsic frequencies ^7– 14^. No one factor, or combination of factors, could fully account for unequal V gene usage in studies considering V germline transcript levels, transcription factor (TF) binding, RSS quality, and the distribution of a variety of epigenetic marks ^7– 9, 11, 15^. Hence, the mechanisms underlying V gene rearrangement frequencies remain to be determined.

Antigen receptor loci are quite large spanning 0.67 Mb- 3.0 Mb and containing up to 100 functional V genes. Chromatin conformational changes in antigen receptor loci are important determinants for long-distance V(D)J recombination events ^6^. Developmental stage-specific contraction of Ig and TCR loci promotes proximity of J-distal V genes with (D-)J segments and generally is thought to facilitate recombination but this has not been formally proven ^16– 19^. Two currently unresolved questions in the formation of the antigen receptor repertoires are (1) what is the molecular basis for locus contraction that is hypothesized to support V->DJ recombination over exceptionally long genomic distances and (2) what underlies the unequal rearrangement potential of individual V genes? Here, we focus on the murine Igh and Igκ loci to address these questions. The molecular principles resulting from these studies may be generally applicable to all antigen receptor loci.

## Locus contraction is a feature of antigen receptor loci

Developmental activation of the Igh locus is a stepwise process that features acquisition of epigenetic modifications, DNase I hypersensitive sites, and the onset of sense and anti-sense transcription ^16, 20– 23^. Additionally, the Igh locus undergoes large-scale locus contraction during development that is detected by using three-dimensional (3D) DNA fluorescence *in situ* hybridization (FISH) methods ^24, 25^.

The early observations by Kosak revealed two fundamental findings regarding the disposition of the Igh locus in the nucleus ^24^. First, the Igh locus is located at the nuclear periphery in non-B cells and relocates to the nuclear center at the pro-B cell stage ^24^ through a process that requires active dislocation from the nuclear lamina ^26^. Second, the Igh locus is in an extended conformation in non-B cells and lymphoid progenitors, whereas both Igh alleles are contracted in pro-B cells, a developmental stage coincident with V(D)J recombination ^24, 25^. These pioneering studies have led to the recognition that all of the large antigen receptor loci undergo developmentally regulated conformational changes before rearrangement at that locus ^24, 25, 27– 30^. The contracted Igh locus in pro-B cells undergoes decontraction at the pre-B cell stage of development to prevent a second round of V _H_-D _H_J _H_ rearrangement on the second Igh allele, presumably aiding allelic exclusion ^28^. Degrees of locus compaction have been inferred from the relationship of inter-probe nuclear distances derived from 3D DNA FISH versus genomic distances and these measurements have limited resolution (100–1000 nm). Consequently, it has been difficult to identify DNA elements that mediate locus contraction.

Igh locus contraction depends on the TFs Pax5, Ikaros, and YY1 ^25, 31, 32^. Loss of Igh locus compaction is correlated with preferential usage of the most D _H_-proximal V _H_ genes ^25, 31, 33^, indicating that spatial access to the more distally positioned V _H_ gene segments has been lost. Although depletion of any of these TFs reduces distal V _H_ rearrangement, chromatin accessibility remains unchanged ^25, 31, 33^. Low-level transcription over V _H_ genes and intergenic regions occurs as the locus is preparing to undergo rearrangement ^34– 37^. The highest level of non-coding RNA (ncRNA) in the Igh locus is found at elements called Pax5-activated intergenic repeats (PAIRs) and these ncRNAs are dependent upon the presence of Pax5 and YY1 ^37, 38^. Although the function of TFs in locus contraction remains speculative, PAIR elements have been suggested to induce long-range chromatin looping by relocating to transcription factories where they associate with the 3′ proximal Eμ-J _H_-D _H_ domain ^37^. However, the molecular mechanism that mediates locus contraction remains unclear.

## The Igh locus is conformationally distinct in pro-B cells

Eukaryotic chromosomes are organized into higher-order spatial configurations of multiple-length scales as determined by using high-resolution chromosome conformation capture (3C)-based approaches and microscopy-based methods, including 3D DNA FISH and live cell imaging ^39– 47^. For example, insulators and enhancers often engage in physical interactions with their target promoters ^48– 51^, indicating that regulatory elements can control distant gene expression through direct long-range molecular contact. However, not all long-range chromatin interactions are directed toward regulating gene expression. For example, intra-chromosomal interactions are required to regulate V(D)J recombination and Ig class-switch recombination (CSR) ^6, 16, 17, 52^. In CSR, the constant (C _H_)-region exons encoding IgM are substituted with a downstream C _H_ gene such that IgM is no longer produced and instead IgG, IgE, or IgA is made in conjunction with the original recombined variable-region exons. CSR is dependent on 3D chromatin architecture mediated by long-range intra-chromosomal interactions between distantly located transcriptional elements ^53– 56^. During V(D)J recombination, antigen receptor genes undergo ordered rearrangement with D _H_-to-J _H_ joining preceding V _H_-to-D _H_J _H_ recombination ^4^. To produce a fully representative Ig repertoire, it is essential that the distal V _H_ genes achieve spatial proximity with the RC and D _H_J _H_ domain. Murre and colleagues have shown that Igh locus topology is best described as a series of three large chromatin loops joined by linkers in pre-pro-B cells but that these loops have intermingled and provide equal access of the D _H_-distal and -proximal V _H_ gene segments with rearranged 3′ D _H_J _H_ in pro-B cells ^57^. The time interval for D _H_J _H_ to gain proximity with a V _H_ gene segment is on the order of minutes, and spatial confinement of topological domains largely regulates first-passage times for chromatin interactions *in vivo* ^58^. Although it is clear that Igh locus conformation is structured, the DNA elements that anchor chromatin looping in support of V(D)J recombination remain largely undefined.

## The Igh and Igκ loci are configured as topologically associating domains

Topologically associating domains (TADs) are megabase sized and represent regions of high-frequency self-interacting chromatin contacts as defined in 3C-based studies ^59, 60^. The organization of interphase chromatin is largely conserved between cell types, especially with regard to TAD boundaries ^44, 50, 61^. Strikingly, the Igh locus is contained within a 2.9-Mb TAD in pro-B cells ^1^.

The murine Igh TAD is partitioned into two highly structured sub-TADs A and C—corresponding to the D _H_-proximal and D _H_-distal V _H_ gene families, respectively—and flank a less structured sub-TAD B that includes the intermediate V _H_ gene segments ^1^ ( Figure 1C). Sub-TADs are zones within a TAD in which chromatin contacts are more frequent than with sites outside the sub-domain, and contacts can be tissue-specific and can contribute to the overall architectural structure of the TAD ^49, 62, 63^.

 V genes can be subdivided into V families based on sequence relatedness and this reflects gene duplication and divergence of primordial V genes. The correspondence of sub-TAD structure with the murine V _H_ gene family distribution profile is striking ( Figure 1C). In the murine Igh locus, V _H_ families tend to be clustered, but in most other antigen receptor loci and in other species, the members of individual V _H_ families generally are interspersed.

The Igκ locus is also contained within a 3.5-Mb TAD which is subdivided into five sub-domains ^2^ ( Figure 2A, B). However, because Vκ gene families are interspersed across the locus, there is no correspondence between Vκ families and sub-TAD structure. One unique feature of the Igκ locus is that about one third of Vκ genes are present in the reverse orientation such that they rearrange to Jκ genes by inversion as opposed to the predominant deletional rearrangement found at other antigen receptor loci. However, there is no correlation between sub-TAD structure and the inversional or deletional orientation of Vκ genes. The conservation of Igκ TAD and sub-TAD structure has not been examined in different cell types.

## Igh TAD conformation is sculpted by developmentally specific chromatin looping

TADs can be thought of as scaffolds for constitutive architectural interactions. Nevertheless, interactions within TADs may vary significantly between cell types or developmental stages and for private enhancer–promoter contacts ^44, 50, 64– 68^. Igh sub-TADs A, B, and C become juxtaposed in pro-B cells via megabase-scale chromatin looping but these contacts are absent in non-B cells ^1^. The loop anchors located in sub-TAD A and C are termed sites I, II, and III ( Figure 1C). Our FISH studies indicated that sites I, II, and III participate in three-way physical contacts in about 32% of pro-B cells and in less than 5% of non-B cells and may functionally create proximity between the distal V _H_ domain with the RC/D _H_/J _H_ region to facilitate efficient access of all V _H_ gene segments for recombination ^1^.

The structure of sub-TAD A is worthy of additional consideration as it contains the Eμ and 3′Eα enhancers, the RC located in the J _H_-D _H_ domain, intergenic control region 1 (IGCR1) (an insulator which will be discussed in detail in sections below), and the proximal V _H_ genes ( Figure 1A). Sub-TAD A becomes modified in pro-B as compared with non-B cells. In non-B cells, Igh sub-TAD A encompasses the proximal V _H_ genes spanning from site I to Eμ ( Figure 1C). In pro-B cells, sub-TAD A becomes subsumed within a larger topological fold that extends from site I to the 3′Eα enhancer ( Figure 1C). The higher-order chromatin structure in pro-B cells may have significant implications for D _H_-proximal V _H_ gene usage during V(D)J recombination.

Several earlier observations have shown that D _H_-proximal V _H_ genes are regulated differently from the rest of the V _H_ genes. Although distal V _H_ gene recombination is reduced in Pax5-, YY1-, and Ezh2-deficient pro-B cells, D _H_-proximal V _H_ genes recombine normally ^25, 31– 33^. Thus, the localization of the D _H_-proximal V _H_ genes within the same conformational sub-TAD as the RC/D _H_/J _H_ region distinguishes them from distal V _H_ genes that lie within sub-TADs B and C.

Pax5 organizes sub-TAD C that spans the distal V _H_J558 gene family. Site III within sub-TAD C fails to associate with sites I and II in Pax5-deficient pro-B cells, thus providing a possible explanation for reduced V _H_J558 rearrangements in Pax5-deficient pro-B cells ^1^. Notably, 14 PAIR elements that were proposed to mediate locus compaction via Pax5 are all situated within sub-TAD C, and PAIR motifs 10 and 11 overlap with site III ^1, 38^. PAIR elements are bound by the TFs Pax5, E2A, and CTCF (CCCTC-binding factor) in pro-B cells ^38^. It is not known whether transcriptional activity at PAIR elements regulates chromatin looping. Our studies provide a potential molecular definition of locus contraction by identifying loop anchor sites that are key mediators of this process.

## CTCF mediates insulator function at TAD boundaries

TAD boundaries are frequently enriched for CTCF binding and CTCF-binding elements (CBEs) ^45, 59, 60, 62, 63^. CTCF is a ubiquitously expressed zinc-finger protein that binds DNA, functions as an insulator in vertebrates ^69^, and plays a key role in chromatin looping ^45, 63, 70, 71^. There is an observed inward or convergent orientation of CBEs flanking TADs ^45, 70, 72, 73^. Insulators were originally defined as genomic elements that act as a barrier to position effects caused by the spreading of chromatin marks and they block enhancer activity ^74, 75^. Although loci situated within TADs are relatively insulated from loci outside the domain, these same elements readily interact with other loci within the same domain. CRISPR/Cas9-mediated rearrangements of TAD boundaries and regulatory elements facilitate or prevent looping interactions with distal regulatory elements ^76– 78^. Acute depletion of CTCF leads to loss of loop domains and impaired regulation of nearby genes through loss of enhancer insulation ^79^.

High-resolution *in situ* Hi-C studies demonstrated that mammalian genomes are partitioned into contact domains ^45^. Contact domains with end points that anchor a loop are referred to as loop domains ^45, 70^. TADs are most frequently loop domains but not all loop domains are TADs. In the context of V(D)J recombination, RAG recombinase activity was shown to be confined to loop domains that are defined by convergent CTCF-bound elements. RAG primarily initiates double-stranded breaks (DSBs) at RSSs within the antigen receptor loci. However, RAG can also initiate low-frequency DSBs at off-target sites that have sequence similarity to RSSs and cause chromosomal rearrangements and translocations ^52, 80– 82^. Notably, when RAG was experimentally directed to chromosomal domains outside of antigen receptor loci, off-target DSBs were confined within loop domains and deletion of convergent CBEs extended the range of RAG activity ^83^.

## CTCF partners with cohesin to mediate chromatin looping

CTCF-based long-range looping interactions are dependent on co-binding with cohesin ^84, 85^. The cohesin complex is thought to form a ring around two CTCF proteins bound to DNA ^85, 86^. Different combinations of architectural proteins may mediate context-specific genomic organization ^63, 87^. Promoter–enhancer interactions are disrupted in embryonic stem cells ^88^ and in thymocytes ^89^ when cohesin is depleted. There is a rich CTCF-cohesin landscape in the Igh locus. One hundred thirty-two sites are bound by CTCF and cohesin and the majority of these are located at a distance of 1 to 32 kb from V _H_ gene segments in the Igh locus ^90, 91^. Strikingly, all of the rearranging D _H_-proximal V _H_ genes are closely paired with CBEs that are located within 68 base pairs (bp) of the RSS ( Figure 1A) ^90^. However, CBEs in the non-rearranging D _H_-proximal V _H_ genes are located more than 1 kb from the RSS in the two most D _H_-proximal V _H_ gene families. As described below, close proximity to the adjacent CBE has functional consequences for these V _H_ genes ^7, 91, 92^. In addition, a cluster of nine CBEs marks the 3′ boundary of the Igh TAD ^92^, and two CBEs located within IGCR1 mark the boundary between the D _H_J _H_ domain and the D _H_-proximal V _H_ genes ( Figure 1B) ^20, 93, 94^. Similarly, the Tcrb and Tcrd loci have CBEs located between the V and J gene segments ^30, 95, 96^. In the Igκ locus, CBEs, termed contracting element for recombination (Cer) and silencer in the intervening sequence (Sis), are located between the V and J gene segments, and many CBEs are found throughout the Vκ domain ( Figure 2A) ^91, 97, 98^.

In the Igh and TCRβ loci, all bound CTCF sites in the V exon domains upstream of the D-J-C-regions are oriented toward them, and the CBEs in D-J-C regions of those loci are oriented toward the V exons. In contrast, the other two large antigen receptor loci (TCRα/δ and Igκ) have more complex patterns with the bound CTCF sites in the V gene portion of each locus found in both orientations ^91^. A role for the CTCF-cohesin complex in Igh locus looping has been suggested by shRNA knockdown studies in pro-B cells demonstrating that the Igh locus is less contracted after CTCF is knocked down ^99^.

## A convergence of loop extrusion and directional RAG tracking?

**Figure 3.**
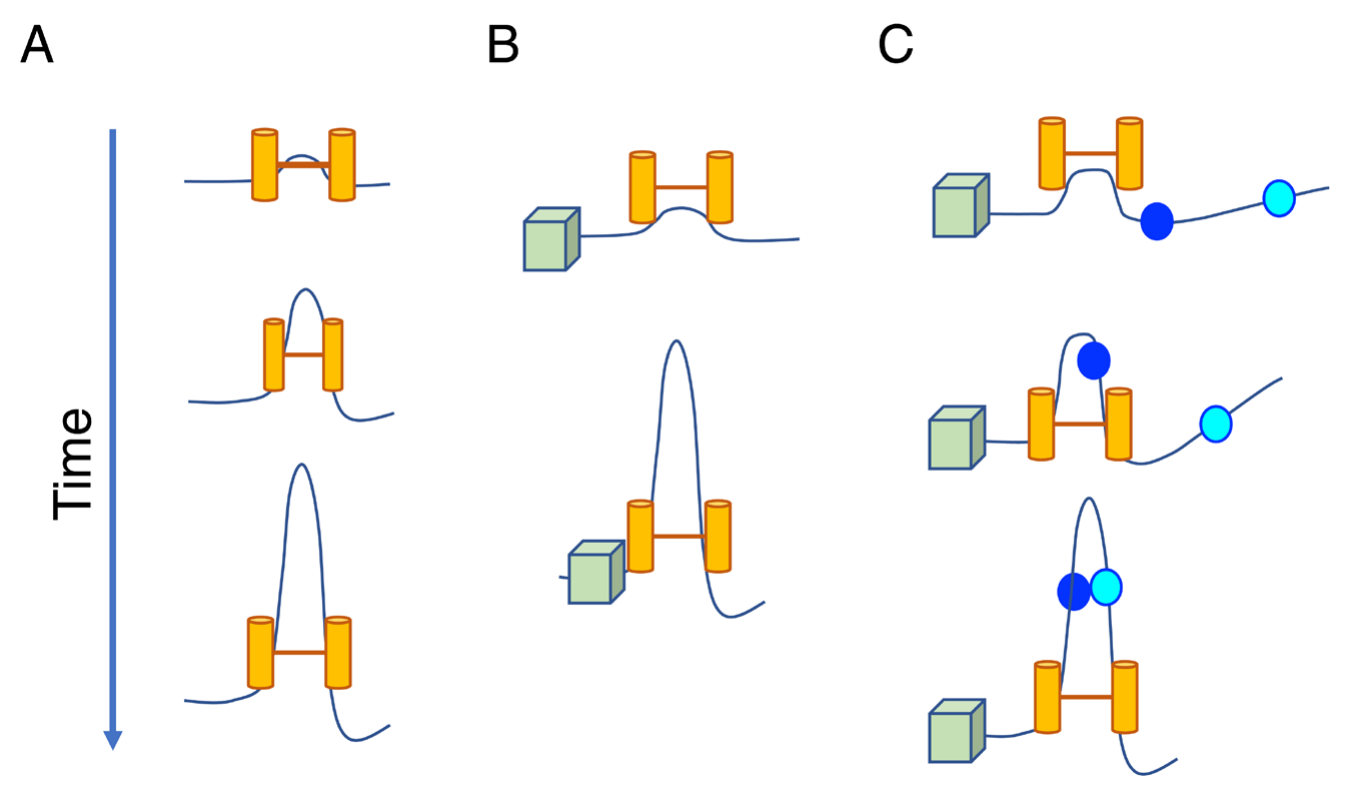
Loop extrusion as a topologically associating domain generating machine. ( **A**) The chromatin fiber extrudes over time through an extruding factor (possibly cohesin; yellow cylinders). ( **B**) A boundary element (possibly CTCF, green cube) can block loop extrusion when the CTCF-binding element is in the proper orientation. It has been proposed that CTCF can block extrusion by one of the cohesin extruding motors while the second motor will be unobstructed and continue to extrude the loop ^102^. ( **C**) Regulatory elements may come into close molecular contact by the process of loop extrusion. These interactions will occur only within a topologically associating domain and in the presence of extrusion blocking elements.

TADs and loop domains have been implicated in regulating gene expression in mammalian cells ^40, 86^, with convergent CBEs in a large subset of cases ^72, 73, 100, 101^. It has been proposed that TADs can be formed by the loop extrusion activity of cohesin ( Figure 3A) ^100, 102^. When cohesin is bound to chromatin, it forms a progressively larger loop until it encounters an obstacle formed by another cohesin or boundary protein including CTCF ( Figure 3B). The association of CTCF with widely separated convergent CBEs may involve cohesin that is halted upon arriving at convergent CTCF-bound loop anchors ^40, 100, 102, 103^. It has been proposed that loop extrusion may also facilitate close-range contacts between regulatory elements, including promoters and enhancers, by bringing them into molecular contact ^40^ ( Figure 3C). Promoter–enhancer interactions may preferentially occur within chromatin domains that are insulated by extrusion blocking factors.

In a situation strikingly analogous to convergent CBE-mediated loop formation, RAG-dependent recombination involves interactions between distant convergent RSSs with the exception of inverted RSSs in some antigen receptor loci. The Alt group has shown that RAG off-target activity within CTCF loop domains spanning 2 Mb depends on orientation-specific RSSs ^83^. It was inferred from DNA sequencing data that RAG can travel directionally from a physiological or ectopically introduced RC within a convergent CBE-based loop domain of megabase size ^83^. Long-range directional exploration by RAG can be blocked by an encounter with cohesin-bound convergent CBE pairs and possibly by other impediments that create chromatin sub-domains within TADs ^83, 104^. Alt and colleagues proposed that RAG complexes bind one RSS and then track along the chromatin fiber in a linear fashion to the next convergent RSS ^82, 104^. Several different topological machine models have been postulated to explain directional cis-guided long-range looping interactions ^40^. It remains unclear whether RAG tracking occurs via loop extrusion or by a mechanistically different activity.

## IGCR1 is an insulator that partitions the D _H_J _H_ domain from V _H_ genes

CTCF has been implicated as a mediator of transcriptional insulation through its ability to participate in chromatin looping ^71^. The striking number and organization of CBEs across antigen receptor loci have led to the proposal for a role of CBE in V(D)J recombination ^93, 99^. The Igh sub-TAD A contains several important looping contacts, including Eμ-IGCR looping interactions in pro-B cells ( Figure 1C) ^93, 105^. IGCR1 contains a pair of divergent CBEs that demarcate the boundary of the RC/D _H_J _H_ domain and function as an insulator that prevents D _H_-to-V _H_ joining prior to D _H_J _H_ rearrangements ^93, 94^. However, the relationship of CTCF-anchored chromatin looping for V _H_:IGCR1 and antigen receptor rearrangement frequency remains unclear.

V _H_ CBEs are convergently oriented with respect to the upstream IGCR1 CBE, and 3′ CBEs are convergently oriented relative to the downstream IGCR1 CBE ^93^ ( Figure 1A). Although CBE-dependent Eμ:IGCR1 looping is prominent in pro-B cells, it is striking that Eμ:V _H_81X contacts are largely undetectable in the wild-type context, indicating that the RC located between Eμ and IGCR1 is sequestered away from all V _H_ genes ^106, 107^. The functional D _H_-proximal V _H_ genes are very closely paired with CBEs ^90^. For example, V _H_81X is the first functional proximal V _H_ gene located about 100 kb from IGCR1 and it is immediately adjacent to a CBE ( Figure 1A). Two new studies have shown that when the IGCR1 CBEs are deleted, Eμ-V _H_81X contacts are newly observed, indicating that IGCR1 CBEs prevent looping interactions between the Eμ-RC/D _H_J _H_ domain and the proximal V _H_ genes ^106, 107^. Strikingly, IGCR1:V _H_81X interactions are dependent on the V _H_81X CBE, as shown by deletion of the V _H_81X-flanking CBE.

Interestingly, the Igκ locus contains the Cer/Sis CBEs in the V-J intervening region ( Figure 2A). Deletion or inversion of Cer leads to preferential usage of Jκ-proximal Vκ genes ^97, 108^, highlighting the importance of convergent CTCF-mediated long-range interactions that facilitate spatial proximity of the distal Vκ with the J segments. Cer/Sis and IGCR1 are similarly located between the V genes and the (D)J genes, and both are involved in mediating chromatin looping. Cer also functions as a transcriptional insulator ^108^.

## Proximal V _H_ gene rearrangement frequencies are determined by CTCF looping

To begin, one might expect that the V _H_5-1 gene segment would be highly used in V _H_-to-D _H_J _H_ rearrangements since it is most proximal to the RC/D _H_J _H_ domain ( Figure 1A). However, despite being paired with a highly conserved RSS, V _H_5-1 is not used in V(D)J recombination. In contrast, V _H_81X (V _H_5-2), the next V _H_ gene segment along the genome, is the most frequently used in V(D)J recombination. The question of why V _H_81X and not V _H_5-1 is used is long-standing. Two groups have explored the relationship between CTCF-mediated chromatin looping and proximal V _H_ gene usage during V(D)J recombination ^106, 107^.

CBEs adjacent to the functional D _H_-proximal V _H_ genes are found within 68 bp downstream of the RSSs ^90^. Mutagenesis analyses have revealed that proximal V _H_ CBEs dramatically influence the frequency of V(D)J rearrangement of that V _H_ gene ^106, 107^. Mutation of the CBE associated with V _H_81X (V _H_5-2) ( Figure 1A) greatly reduced both looping with IGCR1 and its rearrangement frequency and boosted the rearrangement frequency of the next most upstream V _H_ gene, V _H_2-2 ^106^. Genomic editing of the non-functional V _H_5-1 CBE into a functional motif turns this non-rearranging V _H_ gene into the most frequently rearranging gene ^106^ ( Figure 1A). Thus, as discussed below, CBE quality and chromatin looping between IGCR1 and the D _H_-proximal VH gene segments are significant factors determining V _H_ gene usage in V(D)J recombination.

The antigen receptor loci have a much higher density of CTCF sites than the genome overall, making CTCF/cohesin a candidate for forming multiple long-range loops within these loci ^90, 91^. Although it is clear that TAD boundaries are usually formed between convergent CBEs ^45, 72^, relatively little is known regarding the CBE orientation dependence in anchoring chromosome loops within the V domains of Ig loci. All of the bound CBEs in the V _H_ domain are oriented toward the 3′ regulatory region (3′RR) and a single CBE within IGCR1 ( Figure 1A). If CTCF-mediated looping occurs only between convergent CBE, one would predict that the orientation of motifs adjacent to proximal V _H_ genes will be critically required for looping and V(D)J rearrangement. However, when the V _H_81X CBE was inverted, usage of V _H_81X in V(D)J rearrangement was only modestly decreased ^106^, indicating that the orientation specificity inside the V _H_ sub-TADs is not strictly required.

Together, these studies demonstrate that the proximal V _H_ gene CBE’s quality determines looping efficiency with IGCR1 and determines that V _H_ gene’s recombination efficiency. It is noteworthy that most V _H_ and all Vκ genes do not have any CTCF sites in close proximity, in contrast with the location of CBEs for the proximal V _H_ genes ^90, 91^. Thus, for the majority of V genes, CBE-mediated looping with IGCR1 may have a less straightforward impact on V _H_ gene rearrangement frequency.

## Vκ rearrangement frequency is determined by enhancer E88

In addition to long-range loops mediated by CTCF, other long-range loops can be enhancer-mediated. The Igκ locus is encompassed within a TAD that is subdivided into at least five sub-TADs A–E based on Hi-C studies ( Figure 2A) ^2^. We identified a novel enhancer element, E88, which is located close to the boundary separating sub-TADs C and D and which becomes active at the pro-B cell stage prior to V-J rearrangement ( Figure 2A) ^2^. E88 is the major site of interaction with iEκ as detected by 4C analyses in pro-B cells. In pre-B cells, the stage at which V-J recombination occurs, E88 continues to interact strongly with iEκ and also contacts many more sites throughout the locus ( Figure 2B). Strikingly, deletion of E88 results in significant changes in long-range looping interactions and in reduction in rearrangement levels of adjacent Vκ genes ( Figure 2C). Its deletion also results in a modest but consistent reduction of rearrangement of almost all Vκ genes in a 1.5 Mb region surrounding E88 that corresponds to sub-TADs C and D ( Figure 2C). Most Vκ genes that are upstream and downstream of sub-TADs C and D—located in sub-TADs A and B and sub-TADs E, respectively—were modestly increased in relative rearrangement frequency ^2^. Thus, our studies revealed the novel concept that Vκ rearrangement is regulated in a domain-specific manner and suggest that sub-TAD structure has functional ramifications.

## Future questions

Chromatin conformation is now recognized as an important feature regulating gene expression and recombination. Although locus contraction has been a recognized feature of antigen receptor loci for more than 15 years, its underlying molecular mechanism remains largely undefined. Recent studies have provided new insights regarding the convergence of chromatin conformation, TAD and sub-TAD structure, and CTCF-cohesin-mediated looping with V(D)J recombination. These studies revealed that the conformational organization of the Igh, Igκ, and TCRα/δ loci has significant implications for locus contraction and likely influences skewed V gene usage that together affects the composition of the pre-selected repertoires. Going forward, studies focused on the relationship of CTCF- and promoter-enhancer-mediated chromatin looping with locus contraction are likely to provide new insights. Studies designed to clarify the relationship of CTCF-dependent looping and D _H_-distal V gene rearrangement will be important. It is likely that new enhancers, similar to Igκ E88, will be characterized. The influence of individual enhancers on the frequency of individual V gene usage during initial repertoire formation will be important. The emergence of extremely high-resolution DNA FISH is likely to provide additional insights into locus conformation. Finally, studies that determine the extent to which the pre-selected repertoire determines the shape of the peripheral repertoire will yield new insights.

## Abbreviations

3C, chromosome conformation capture; 3D, three-dimensional; bp, base pairs; C, constant; CBE, CTCF-binding element; Cer, contracting element for recombination; CSR, class-switch recombination; CTCF, CCCTC-binding factor; D, diversity; DSB, double-stranded break; FISH, fluorescence *in situ* hybridization; Ig, immunoglobulin; IGCR1, intergenic control region 1; IgH, immunoglobulin heavy chain; J, joining; ncRNA, non-coding RNA; PAIR, Pax5-activated intergenic repeat; RC, recombination center; RSS, recombination signal sequence; Sis, silencer in the intervening sequence; TAD, topologically associating domain; TCR, T-cell receptor; TF, transcription factor; V, variable
